# Moving Knowledge Acquisition From the Lecture Hall to the Student Home: A Prospective Intervention Study

**DOI:** 10.2196/jmir.3814

**Published:** 2015-09-28

**Authors:** Tobias Raupach, Clemens Grefe, Jamie Brown, Katharina Meyer, Nikolai Schuelper, Sven Anders

**Affiliations:** ^1^University Medical Centre GöttingenClinic for Cardiology and PneumologyGeorg-August University GöttingenGöttingenGermany; ^2^Health Behaviour Research CentreDepartment of Epidemiology & Public HealthUniversity College LondonLondonUnited Kingdom; ^3^Department of Clinical, Educational and Health PsychologyUniversity College LondonLondonUnited Kingdom; ^4^University Medical Centre GöttingenClinic for Haematology and OncologyGeorg-August University GöttingenGöttingenGermany; ^5^University Medical Centre Hamburg-EppendorfDepartment of Legal MedicineHamburgGermany

**Keywords:** knowledge, lecture, medical education, podcast, retention

## Abstract

**Background:**

Podcasts are popular with medical students, but the impact of podcast use on learning outcomes in undergraduate medical education has not been studied in detail.

**Objective:**

Our aim was to assess the impact of podcasts accompanied by quiz questions and lecture attendance on short- and medium-term knowledge retention.

**Methods:**

Students enrolled for a cardio-respiratory teaching module were asked to prepare for 10 specific lectures by watching podcasts and submitting answers to related quiz questions before attending live lectures. Performance on the same questions was assessed in a surprise test and a retention test.

**Results:**

Watching podcasts and submitting answers to quiz questions (versus no podcast/quiz use) was associated with significantly better test performance in all items in the surprise test and 7 items in the retention test. Lecture attendance (versus no attendance) was associated with higher test performance in 3 items and 1 item, respectively. In a linear regression analysis adjusted for age, gender, and overall performance levels, both podcast/quiz use and lecture attendance were significant predictors of student performance. However, the variance explained by podcast/quiz use was greater than the variance explained by lecture attendance in the surprise test (38.7% vs 2.2%) and retention test (19.1% vs 4.0%).

**Conclusions:**

When used in conjunction with quiz questions, podcasts have the potential to foster knowledge acquisition and retention over and above the effect of live lectures.

## Introduction

In recent years, there has been increasing interest in the use of podcasts as means of knowledge transmission. In broad terms, podcasts can generally be described as audio and/or video files that can be played back on various electronic devices including tablets and smartphones. In fact, the word “podcast,” first used in 2004, is a portmanteau created from the name of one particular device (the iPod) and the word “broadcast.” There is no uniform consensus as to what format or content is required for an electronic source to be called a “podcast.” As a consequence, anything from straight-forward recordings of lectures or conference presentations to complex animated films can be referred to as podcasts. However, some authors have used the term “vodcast” to describe online material containing videos [1] and “enhanced podcast” for audio material supplemented with still images [2]. One common feature of all these formats is that they can be used in an asynchronous manner (ie, at any time, independent of lecture hours).

Within 10 years of their invention, technologies to capture lectures and make them available to students have been embraced by medical teachers involved in both undergraduate and continuing medical education. At the same time, both massive open online courses [3] and scholarly journals [4] now offer a wide range of options to view or listen to material online. User satisfaction is generally high [5], but there is a paucity of data linking podcast use to actual learning outcome. This is in contrast with recent calls for medical school lectures to be moved to online platforms altogether so that classroom time may be used for more efficient teaching activities [6,7]. The underlying assumption is that students viewing course material in preparation of a lecture will retain the content. To our knowledge, this hypothesis has not been tested so far.

A PubMed search combining “medical education” with the terms “podcasts,” “lecture video,” “online lecture,” or “streaming lecture” (search date March 6, 2014) yielded 357 unique citations, and 6 additional articles were identified from reference lists and by contacting experts in the field. Only 78 out of these 363 papers had a specific focus on podcasts. Only 55 of these presented original data, and about half of these (n=27) were related to undergraduate medical education. While half of these (n=13) just reported usage patterns and student satisfaction with podcasts, only 14 original articles assessed the association between podcast use and learning outcome (6 randomized controlled trials, 7 prospective studies, and 1 retrospective analysis). Notably, none of these studies addressed podcast use for preparatory purposes. Instead, podcasts were used to either completely replace or supplement live lectures. In summary, there is currently no scientific data on the effectiveness of using podcasts to stimulate student learning prior to attending a lecture.

The aims of this study were to assess the impact of preparatory podcast use in conjunction with quiz questions versus lecture attendance on short-term and medium-term knowledge retention, and identify significant predictors of short-term and medium-term knowledge retention.

It was hypothesized that students engaging with the material presented in podcasts and submitting answers to quiz questions would retain significantly more knowledge than students not using podcasts. With regard to the second study aim, it was hypothesized that podcast/quiz use would be at least as effective in promoting short-term and medium-term knowledge retention as lecture attendance.

This study did not address any specific psychological framework underlying a potential effect of podcast/quiz use. Instead, it focused on effects elicited by one particular teaching intervention (ie, podcasts and quiz questions) in a “real-world” educational setting.

##  Methods

### Study Design

This study was conducted at Göttingen Medical School. Like most German medical schools, it offers a 6-year undergraduate curriculum comprising 2 preclinical years and 3 clinical years, followed by a practice year. This prospective trial included a cohort of fourth-year medical students who were enrolled in a 6-week cardio-respiratory module in winter term 2013/14. In the preceding summer term, all 37 lectures held during the 6-week module had been recorded using Camtasia Studio 7 (TechSmith). The resulting videos featured the presentation slides used and the lecturer’s voice (duration: 35-45 minutes; format: MP4). Following the summer term 2013, the material was reviewed, and the best 10 lecture recordings with regard to sound and image quality were selected to be used in this study. Lecturers were asked to identify key aspects with particular relevance for general internal medicine and to draft free-text questions addressing that content (Table 1).

In winter term 2013/14, students enrolled in our module were provided with online access to the 10 selected videos for a period of 7 days before the respective live lectures. A free-text quiz question was linked to each podcast, and students were invited to submit their answers via email until the night before the live lecture. On the day of the lecture, the principal investigator (TR) revealed the correct answer to the entire class and also projected a 1-minute clip from the podcast containing the answer. He then raffled a book voucher (€20) among all students who had submitted a correct answer.

**Figure 1:**
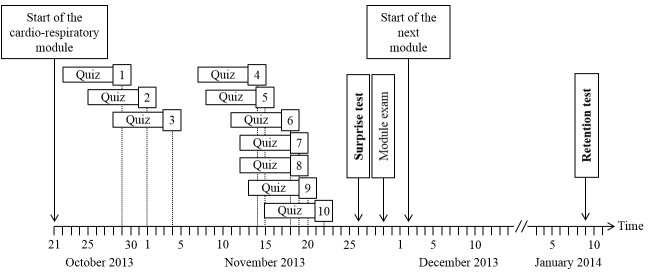
Study outline (numbers in boxes correspond to the 10 lectures used for this study, and vertical dotted lines indicate the date on which live lectures were held). For each lecture, podcasts were available over a period of 7 days leading up to the live lecture. During this time, students were invited to submit their quiz answers.

As part of an e-learning session in the final week of the module, students were invited to complete the same 10 quiz questions that had been provided with the podcasts (surprise test). In this session, students were also asked to indicate which lectures they had attended. In order to assess long-term retention, students were invited to answer the same 10 questions again 2 months later in an unannounced retention test. The study outline is summarized in Figure 1.

**Table 1:** Key aspects covered in podcast lectures and student performance in quizzes as well as in the surprise and retention tests.

Lecture theme	Key aspect	Students with a correct quiz answer n (%)	Students with a correct answer in surprise test n (%)	Students with a correct answer in retention test n (%)
Item 01: Chronic heart failure	Side effects of spironolactone are more pronounced in routine care than in clinical trials due to a lack of potassium monitoring in routine care.	15 (22.4)	13 (19.4)	15 (22.4)
Item 02: Cardiogenic shock	An increase in cardiac output without use of inotropic drugs can be achieved by reducing cardiac afterload.	16 (23.9)	34 (50.7)	34 (50.7)
Item 03: Aortic stenosis	Hallmark symptoms: exertional shortness of breath, angina, and syncope; carotid pulse: prolonged upstroke time.	17 (25.4)	9 (13.4)	11 (16.4)
Item 04: Pacemaker therapy	Effect of placing a magnet over the device: inhibition of shock therapy while pacemaker activity is maintained.	8 (11.9)	21 (31.3)	17 (25.4)
Item 05: Lung function testing	An inhalation test for bronchial hyper-reactivity can be performed only if bronchial obstruction is ruled out in a baseline test.	13 (19.4)	22 (32.8)	23 (34.3)
Item 06: Chronic obstructive pulmonary disease	Neuro-humoral activation is a potential link between intra- and extra-pulmonary manifestations of the disease.	5 (7.5)	14 (20.9)	6 (9.0)
Item 07: Inhaled steroids for asthma	The key to reducing side effects of inhaled steroids was the invention of drugs with high first-pass metabolism.	13 (19.4)	20 (29.9)	5 (7.5)
Item 08: Obstructive sleep apnea	Alcohol intake before going to bed prolongs apneas and causes more pronounced oxygen saturation during sleep.	11 (16.4)	20 (29.9)	16 (23.9)
Item 09: Antibiotics for pneumonia	Ciprofloxacin monotherapy is not recommended as this drug does not target *Streptococcus pneumonia*.	12 (17.9)	27 (40.3)	29 (43.3)
Item 10: Pulmonary fibrosis	New drugs can be assumed to reduce mortality only if this is tested as a primary end point in a randomized trial.	8 (11.9)	15 (22.4)	15 (22.4)

### Student Enrollment and Data Collection

Both the surprise test and the retention test were timed to coincide with scheduled e-learning activity in our institution’s computer facilities. Both tests were unannounced in order to avoid confounding by specific preparation, and a time limit of 15 minutes was set for the completion of the 10 quiz questions in both tests. At the beginning of each e-learning session, the study rationale was explained and students were asked to provide written consent to have their data analyzed for study purposes. A total of 10 book vouchers (€20) were raffled among all participants at both the surprise test and the retention test, regardless of test performance.

Questionnaires were created with EvaSys (Electric Paper). In the surprise test, students were asked to provide their age and gender and to indicate whether they had attended each of the 10 lectures and which podcasts they had watched. The number of quiz answers submitted during the module was derived from the emails sent to the module’s administrative staff. The potential impact of recall bias and/or podcast use without answering quiz questions was assessed by comparing the number of students indicating they had watched a particular podcast with the number of students who had submitted an answer to the corresponding quiz question. In order to adjust the analyses for student performance levels, the percent score achieved by each student in the summative end-of-module examination was also obtained (Figure 1). This examination consisted of 25 multiple choice questions addressing factual knowledge on cardiology and pneumology but specifically excluding the content covered by quiz questions as the latter focused on more complex aspects while multiple choice questions were designed to assess basic factual knowledge.

### Marking of Quiz Answers

The marking procedure was identical for all three time points (during the module—only students who had submitted an answer via email; surprise test and retention test—all students entering data and consenting to have their data analyzed). After agreeing on corrects answers, 2 raters (TR and CG) independently marked all answers as correct (1) or incorrect (0). Inconsistencies were resolved by discussion. In addition to marking each single question, a sum score (0-10) was calculated, reflecting student attainment in quizzes throughout the module, in the surprise test, and in the retention test.

### Statistical Analysis

Unique student identifier codes were used to merge data collected in the surprise and retention tests as well as examination results and data on podcast/quiz use. Data analysis was performed using SPSS Statistics 21 (IBM Corporation). Inter-rater agreement of the marking procedure was assessed by calculating kappa and internal consistency of both tests was assessed by calculating Cronbach alpha.

In order to assess the impact of podcast/quiz use and lecture attendance on short-term and medium-term knowledge retention, the percentage of students providing a correct answer to each question in the surprise and retention tests was calculated. Proportions of students who had/had not submitted a correct quiz answer during the module and those who had/had not attended the corresponding lecture were compared by chi-square tests. Multivariate logistic regression models were run for each of the 10 items with the answer in the surprise/retention test as the dependent variable and controlling for age, gender, and percent score in the end-of-module examination. The comparison between podcast/quiz use and no podcast/quiz use was also adjusted for lecture attendance. Likewise, podcast/quiz use was adjusted for when assessing the impact of lecture attendance on test performance.

A multivariate linear regression analysis was run to identify significant predictors of short-term and medium-term knowledge retention. The dependent variable was the sum score in the surprise/retention test. Age, gender, and percent score in the end-of-module examination as well as the number of submitted correct quiz answers and the number of lectures attended during the module were entered as independent variables.

Group comparisons were performed using chi-square tests (dichotomous variables) and *t* tests (continuous variables). Results of descriptive analyses are presented as percentages and mean with standard deviation (SD), as appropriate. Results of linear regression analyses are reported as unadjusted and adjusted beta values (95% confidence interval) and as the amount of variance explained. Significance levels were set to .05.

### Ethical Approval

The local Institutional Review Board (application number 13/12/13) waived ethical approval as the study protocol was not deemed to represent biomedical or epidemiological research. Study participation was voluntary, and all participants signed an informed consent form before entering the study.

## Results

### Response Rate and Participant Characteristics

Of 130 students enrolled in the module, 126 gave written consent to have their data analyzed for this study. Only students with complete data in both the surprise and the retention tests and the end-of-module examination were included in the final analysis. A total of 101 students attended both tests, but 3 of these did not take the end-of-module examination and another 31 failed to provide complete information on lecture attendance. Thus, complete data of 67 students (24.2 years [SD 2.9]; 39 female) were available. Of these, 34 had submitted at least one correct answer during the module (mean 3.5 [SD 2.6]). On average, students had attended 7.8 (SD 2.3) live lectures. The percentage of students who recalled watching the podcast among those who had submitted a quiz answer was over 80% for all items, suggesting podcast use was not hugely underreported. On the contrary, the proportion of students who had not submitted a quiz answer among those who recalled watching the podcast ranged from 20% to 50%.

### Inter-rater Agreement, Item Characteristics, and Results of the Surprise and Retention Tests

Inter-rater agreement for quiz questions and the surprise and retention tests were acceptable (kappa values were .86, .80, and .90, respectively). Cronbach alpha was .68 and .65 in the surprise and retention tests, respectively. The mean number of correct answers in these tests were 2.9 (SD 2.3) and 2.6 (SD 2.0), respectively. As shown in Table 1, performance in all test items was low to moderate. For example, one-third of students were aware that ruling out bronchial obstruction in a baseline lung function test is a prerequisite for bronchial hyper-reactivity testing and only 1 in 5 students displayed adequate knowledge on how to interpret clinical trial reports.

### Impact of Podcast Use and Lecture Attendance on Test Performance

**Figure 2:**
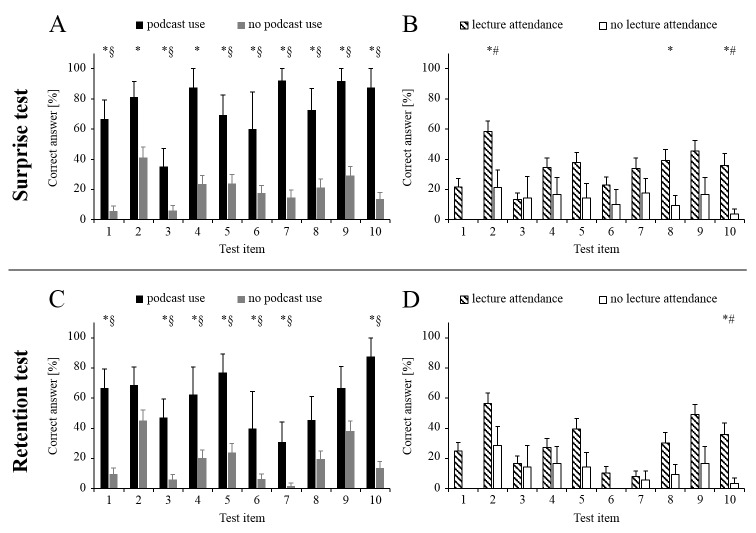
Student performance in the surprise and retention tests (columns represent the percentage of students providing a correct answer; error bars represent standard errors). * P<.05 for direct comparison (chi-square test); § P<.05 in a logistic regression adjusted for age, gender, exam performance, and lecture attendance; # P<.05 in a logistic regression adjusted for age, gender, exam performance, and podcast use.

Figure 2 presents student performance in the surprise and retention tests as a function of podcast use and lecture attendance.

In the surprise test, podcast use was associated with significantly better knowledge on all test items while such associations with lecture attendance were observed for only 3 items. Similarly, podcast use enhanced knowledge in the retention test for 7 items while there was no such effect of lecture attendance for 9 of 10 items. Adjusting for age, gender, and examination performance attenuated the associations, but the pattern of results was unchanged.

In a sensitivity analysis, percentages of correct answers in the surprise and the retention tests were calculated separately for students who (Group 1) had submitted a quiz answer and recalled having watched the corresponding podcast and students who (Group 2) recalled having watched the podcast but had not submitted a quiz answer. With one exception (Item 2), test performance in the second group was similar to the performance of students who had neither submitted a correct answer nor watched the podcast. The proportion of correct answers in Group 2 was less than half of that observed in Group 1 and was 0% for 3 items in the surprise test and 4 items in the retention test (data not shown).

### Predictors of Short-Term and Medium-Term Knowledge Retention

Results of the linear regression analyses are reported in Table 2. Podcast/quiz use and lecture attendance were significant predictors of student performance in both tests. However, the variance explained by podcast/quiz use was greater than the variance explained by lecture attendance in the surprise test (38.7% vs 2.2%) and retention test (19.1% vs 4.0%).

**Table 2:** Predictors of student performance in the surprise and retention tests (*R* ^2^, variance explained).

Variables		Unadjusted beta (95% CI)	Adjusted beta (95% CI)	*R* ^2^
**Sum score in the surprise test**				
	Female gender	0.95 (-0.16 to 2.06)	0.25 (-0.44 to 0.94)	.003
	Age in years on the first day of the module	-0.08 (-0.28 to 0.11)	-0.05 (-0.17 to 0.07)	.004
	Percent score in the module examination	0.08 (0.04-0.13)	0.06 (0.03-0.09)	.091
	Number of correct quiz answers submitted during the module	0.65 (0.50-0.81)	0.60 (0.46-0.73)	.387
	Number of lectures attended during the module	0.39 (0.17-0.61)	0.15 (0.004-0.30)	.022
**Sum score in the retention test**				
	Female gender	0.95 (-0.05 to 1.95)	0.33 (-0.47 to 1.13)	.006
	Age in years on the first day of the module	-0.17 (-0.34 to 0.01)	-0.13 (-0.27 to 0.02)	.026
	Percent score in the module examination	0.07 (0.03-0.11)	0.05 (0.01-0.08)	.062
	Number of correct quiz answers submitted during the module	0.44 (0.27-0.61)	0.38 (0.22-0.54)	.191
	Number of lectures attended during the module	0.37 (0.17-0.57)	0.19 (0.02-0.36)	.040

## Discussion

### Principal Findings

This is the first study to examine the impact of podcast use in conjunction with quizzes prior to lecture attendance on knowledge acquisition and retention in undergraduate medical students. Students who engaged with the material before the lecture displayed improved short-term and medium-term retention, regardless of whether they also attended the lecture. The impact of lecture attendance on knowledge retention was considerably weaker despite the correct answers and the decisive part of the podcast being presented to all students in the lecture hall. The most likely explanation for our finding is that—just like interaction during a live lecture [8]—the questions provided with preparation podcasts stirred student alertness, thus facilitating learning [9]. This notion is supported by the results of the sensitivity analysis indicating that watching podcasts without submitting an answer to the corresponding quiz question did not result in improved short-term or medium-term retention. It might be hypothesized that a similar effect could have been observed for lecture attendance if students had been asked to pay attention to a specific detail during the lecture and submit the answer to a related question afterward. However, according to the rationale outlined earlier, one potential use of podcasts could be to partially move the process of knowledge acquisition from the lecture hall to the preparation phase, thereby enabling teachers and learners to explore new and better ways to spend classroom time [10].

### Research Context

There has been some debate about the usefulness of podcasts in medical education. While some authors regard them as “toys” [11] and have called for more research into their actual effectiveness, others have argued that students can benefit from exploring novel technologies even in the absence of randomized controlled trials demonstrating their effectiveness [12].

The 14 published reports on the impact of podcast use on learning outcomes in undergraduate medical students vary considerably with regard to study design and outcome measure used. One retrospective analysis detected a small effect of podcast availability on national licensing examination scores that coincided with a national trend for better examination scores [13]. While 2 of the 7 prospective trials found no effect of supplemental podcasts on test scores [14,15], others did find an effect [16,17]. However, some of these effects were either assessed at a very early follow-up (ie, 5 days [18]) or confined to specific student populations, for example, non-native speakers [19]. In one study, students viewing more lectures were even found to score lower in a consecutive examination [20]. Of the 6 randomized trials published so far, 3 [21-23] found a significant effect of podcast use on student examination performance, whereas the other 3 did not [24-26].

In our study, podcasts were used neither to replace nor supplement lectures but as a preparatory tool. In this regard, our results provide some suggestions on *how* this technology might be used to improve learning outcome [27] (as opposed to assessing *whether* it should be used at all [28]). When combined with quiz questions, the provision of podcasts led to a more favorable learning outcome than lecture attendance itself, and this effect was sustained and robust in the adjusted analysis. Given the relatively low uptake observed in our study and previous studies [20], one potential practical implication of our findings could be making the completion of a “preparatory podcast/quiz task” a requirement for course attendance.

### Strengths and Limitations

Whereas many previous outcome studies assessed the association between podcast use and overall examination scores, the surprise and retention tests we used were created specifically for this study, and we made every effort to align test questions to the content taught in podcasts and lectures. Inter-rater agreement and internal consistency of the surprise and retention tests were acceptable, but mean scores in both tests were surprisingly low. One potential explanation for this is that these tests were formative in nature, and students might not have made full efforts to achieve a maximum number of correct answers. However, this should apply to all students (regardless of podcast/quiz use and lecture attendance), and using summative examinations would have had a confounding effect likely to mask any real effect of podcast/quiz use on knowledge levels [29,30]. Another explanation for the low overall scores observed in this study is that quiz questions were related to complex clinical content that—despite being highly relevant for medical practice—is not usually being covered in undergraduate medical textbooks. Moreover, students at our university are not used to open-ended questions as most end-of-course examinations still consist of multiple choice questions. The small amount of variance in surprise and retention test scores explained by the summative multiple choice examination (9.1% and 6.2%, respectively) can be taken as evidence of discriminant validity in that the study-related tests featuring open-ended questions assessed different types of knowledge than the multiple choice questions presented in the end-of-module examination.

One particular strength of our study was the ability to disentangle the effects of podcast/quiz use and lecture attendance in the adjusted analyses presented in Table 2. These data suggest that following podcast use and submitting a correct answer, attending the live lecture had only limited additional benefit in terms of learning outcome. We cannot rule out the possibility that students prepared for lectures with material other than podcasts and/or quiz questions. However, given the marked performance differences between podcast/quiz users and nonusers, any effect of additional preparation would be either confined to podcast/quiz users or too small to detect in students not using podcasts/quizzes.

We excluded a large number of students due to missing information on lecture attendance. This led to a student sample favoring slightly younger (24.2 [SD 2.9] vs 25.4 [SD 2.9]; *P*=.019) and slightly higher-performing students (end-of-module exam scores: 78.6% [SD 11.3] vs 72.8% [SD 13.8]; *P*=.012). The impact of these variables on our results within the final study sample was accounted for by adjusting our analyses accordingly. In addition to selection bias, recall bias is another potential threat to the validity of our findings. However, a great majority of students who had submitted a quiz answer also recalled having watched the corresponding podcast, rendering underreporting of podcast use unlikely. It might be hypothesized that lectures are in fact effective in helping students to acquire and retain knowledge. In order to artificially increase the effect of podcast/quiz use over that of lecture attendance, podcasts users would have had to systematically underreport lecture attendance. However, this was not the case as students submitting at least one correct quiz answer indicated to have attended significantly more lectures than students not submitting any answer: 8.7 (SD 2.0) versus 7.1 (SD 2.5); *P*=.006. In addition, there was a positive correlation between lecture attendance and podcast use (*r*=.252; *P*=.039), hence the need to control for lecture attendance in the analysis of podcast effectiveness and vice versa.

An alternative approach to addressing our research question would have been to conduct a randomized controlled trial. Although this would have yielded higher internal validity, we doubt that we would have been able to restrict podcast use to a specific student group. The aim of this trial was not to test learning processes induced by the availability of podcasts and quiz questions. Instead, this study assessed the effect of one particular teaching intervention in the “real world” of undergraduate medical education. With regard to generalizability, our findings will need to be replicated in other settings. At the same time, there is no reason to believe that using podcasts supplemented with quiz questions as tools to stimulate student learning would be completely ineffective if implemented in a different medical school.

### Conclusions

When used in conjunction with quiz questions, lecture podcasts have the potential to foster knowledge acquisition and retention over and above the effect of live lectures. Our findings might help pave the way to move knowledge acquisition from the lecture hall to the preparatory phase, thereby freeing up valuable lecture time for more effective learner-teacher interactions.
